# Regulation of Human Trophoblast GLUT1 Glucose Transporter by Insulin-Like Growth Factor I (IGF-I)

**DOI:** 10.1371/journal.pone.0106037

**Published:** 2014-08-26

**Authors:** Marc U. Baumann, Henning Schneider, Antoine Malek, Vidya Palta, Daniel V. Surbek, Ruth Sager, Stacy Zamudio, Nicholas P. Illsley

**Affiliations:** 1 Departments of Obstetrics and Gynecology, Inselspital, University of Berne, Berne, Switzerland; 2 Department of Obstetrics, Gynecology and Women’s Health, New Jersey Medical School, Newark, New Jersey, United States of America; 3 Center for Abnormal Placentation, Department of Obstetrics and Gynecology, Hackensack University Medical Center, Hackensack, New Jersey, United States of America; Tohoku University, Japan

## Abstract

Glucose transport to the fetus across the placenta takes place via glucose transporters in the opposing faces of the barrier layer, the microvillous and basal membranes of the syncytiotrophoblast. While basal membrane content of the GLUT1 glucose transporter appears to be the rate-limiting step in transplacental transport, the factors regulating transporter expression and activity are largely unknown. In view of the many studies showing an association between IGF-I and fetal growth, we investigated the effects of IGF-I on placental glucose transport and GLUT1 transporter expression. Treatment of BeWo choriocarcinoma cells with IGF-I increased cellular GLUT1 protein. There was increased basolateral (but not microvillous) uptake of glucose and increased transepithelial transport of glucose across the BeWo monolayer. Primary syncytial cells treated with IGF-I also demonstrated an increase in GLUT1 protein. Term placental explants treated with IGF-I showed an increase in syncytial basal membrane GLUT1 but microvillous membrane GLUT1 was not affected. The placental dual perfusion model was used to assess the effects of fetally perfused IGF-I on transplacental glucose transport and syncytial GLUT1 content. In control perfusions there was a decrease in transplacental glucose transport over the course of the perfusion, whereas in tissues perfused with IGF-I through the fetal circulation there was no change. Syncytial basal membranes from IGF-I perfused tissues showed an increase in GLUT1 content. These results demonstrate that IGF-I, whether acting via microvillous or basal membrane receptors, increases the basal membrane content of GLUT1 and up-regulates basal membrane transport of glucose, leading to increased transepithelial glucose transport. These observations provide a partial explanation for the mechanism by which IGF-I controls nutrient supply in the regulation of fetal growth.

## Introduction

Glucose, a primary substrate for fetal growth, is transported across the human placenta via the GLUT1 glucose transporter that is found in both the microvillous and basal membranes of the syncytiotrophoblast barrier layer. GLUT1 glucose transporters are asymmetrically distributed, being several fold higher on the microvillous (maternal facing) than basal (fetal-facing) membrane [1], [2]. There is strong evidence that the basal membrane is the rate-limiting step in transplacental glucose transport [3], thus changes in basal membrane glucose transporter expression will have significant consequences for the maternal-to-fetal transport of glucose and for fetal growth. The importance of GLUT 1 in fetal growth is clear. GLUT1 expression increases over the latter half of gestation, concurrent with the increased rate of fetal growth in the third trimester [1]. GLUT1 transporter expression is increased in the basal membrane in diabetic pregnancies [4], [5], while a decrease in basal membrane GLUT1 expression has been shown in altitude-induced hypoxia, concomitant with reduced fetal growth [6]. In addition to the asymmetric distribution of the GLUT1 glucose transporters, varied rates of glucose consumption in syncytiotrophoblast cells have been shown to be an important factor regulating directional flux of glucose from the maternal to the fetal circulation [7].

Although these changes in GLUT1 expression are important for fetal growth, the regulatory mechanisms controlling these changes have yet to be elucidated. Our prior work has focused on factors associated with the aberrant fetal growth observed in conditions such as fetal growth restriction or macrosomia [4], [8]. In this report we have investigated the role of insulin-like growth factor-I (IGF-I) in regulating syncytial GLUT1. There is evidence to support the idea that both maternal and fetal IGF-I can regulate fetoplacental growth. Multiple studies, including those examining the extremes of birth weight in both normal and pathological conditions indicate that maternal and fetal IGF-I levels are correlated with birth weight [9]–[14]. Moreover natural experiments in the human [15] or experimental manipulations in animals have shown that changes in fetal growth are associated with alterations in IGF-I [16]–[18].

Despite the evidence demonstrating a role for IGF-I in fetoplacental growth, there is an absence of information on the specific mechanisms by which this interaction might be mediated. One possible mechanism is the regulation by maternal and/or fetal circulating IGF-I of the trans-syncytial nutrient fluxes mediated via glucose, amino acid and other transporters. GLUT1 expression is regulated by IGF-I in a variety of tissues [19]–[22]. The type 1 IGF receptor is present on both the microvillous and basal membrane of the syncytiotrophoblast [23], [24]. It is possible therefore that IGF-I, through the modulation of glucose transporter expression on either the maternal or fetal side of the syncytium, could have significant effects on fetal growth. We hypothesized that GLUT1 protein expression on the basal membrane of trophoblast cells is upregulated by IGF-I. In this report we investigated the effects of IGF-I on the expression of GLUT1 glucose transporter protein and glucose transport function in trophoblast cells, placental explants and a placental dual perfusion model.

## Methods and Materials

### Placental tissue/ethics statement

Human placental tissue was obtained from normal, term singleton pregnancies following non-laboring, elective Cesarean section. Written, informed consent was obtained using a protocol approved by the New Jersey Medical School Institutional Review Board (explant and cell studies) or a protocol approved by the ethical committee of the Canton of Berne, Switzerland (perfusion studies). Subjects were excluded if there was evidence of fetal anomalies, intrauterine growth restriction, diabetes, hypertension, anemia, tobacco or drug use or other medical or obstetric complications.

### Placental explants

Villous tissue was washed in cold PBS and dissected into 3–5 mm fragments immediately following delivery. Dissected fragments were maintained in ice-cold PBS during the dissection period. Fragments (∼3–5 mm, total of 2 g) were incubated in T75 tissue culture flasks in 40 mL of DMEM containing 5 mM glucose, 0.5% BSA and 1% penicillin/streptomycin/gentamycin for 3 hr. on an oscillating shaker in a humidified CO_2_ incubator. Following this incubation period, IGF-I (200 ng/mL) was added to the experimental samples. The incubation for control (no addition) and experimental samples was then continued for another 18 hr. After this incubation, the fragments were pelleted by centrifugation at 500×g for 2 minutes and used for the preparation of syncytial microvillous and basal membranes (see below).

### Placental perfusion

Perfusion was performed as described previously [25], [26]. Immediately following elective cesarean section cannulae were placed in the artery and the vein of a placental lobule (cotyledon) to establish the fetal circulation; four needles located within the intervillous space served to provide the maternal circulation. The maternal and fetal circulations were perfused with a medium composed of NCTC 135 and Earle’s buffered salt solution (2∶1) containing 10 g/L dextran 40, 40 g/L human serum albumin and 2500 IU/L heparin. The maternal perfusate was equilibrated with an atmospheric gas mixture and contained 6 mM D-glucose. The fetal circulation was gassed with 95% N_2_/5% CO_2_ and contained 3 mM D-glucose. The intervillous space (maternal circulation) was perfused with medium containing [^3^H] 3-O-methyl-D-glucose and [^14^C] L-glucose (0.064 and 0.032 µCi/mL, respectively) for periods of 30 minutes (open fetal circulation) to establish rates of transfer from the maternal to fetal circulation. For all placentas used in the perfusion studies, tissue samples were taken from placental lobules that were not used for perfusion. These are identified as “immediate control” samples. Villous tissue from immediate controls was prepared by removal of a decidual layer and the chorionic plate. This tissue was then washed in ice cold 0.9% saline and utilized for the preparation of syncytial microvillous and basal membranes. Following termination of the perfusion, the perfused lobule was excised and villous tissue was prepared in the same manner to yield control or experimental (IGF-I treated) samples.

### Syncytial membrane preparation

Washed villous tissue or previously incubated villous tissue fragments were used to prepare syncytial microvillous and basal membranes using a previously described method [4].

### BeWo cells

BeWo cells (b30 subclone, obtained from Dr. K. Audus, University of Kansas [27]) were cultured in DMEM/F12 containing 10% FBS. Cells used for the measurement of GLUT1 protein expression were plated in 6-well culture plates and incubated in DMEM/F12/FBS until the cultures were 70–80% confluent. Cells were then switched to serum-free DMEM containing 5 mM glucose and 0.5% BSA for 24 hr. prior to incubation in the same medium without or with IGF-I (200 ng/mL). After incubation cells were washed×2 with cold PBS, extracted with RIPA buffer and extracts were frozen at −80°C until analysis. In the time course experiments where the incubation period extended to 48 hr., medium and IGF-I were replaced after 24 hr. Transepithelial glucose transfer and glucose uptake experiments in BeWo choriocarcinoma monolayers were performed as described previously [3], including measurements of transepithelial electrical resistance to ensure presence of a monolayer. Prior to measurement, growth medium was replaced by DMEM/0.5% BSA containing 5 mM glucose for 24 hr. followed by a further 24 hr. incubation performed in the presence or absence of IGF-I (200 ng/mL) added to both apical and basal reservoirs. Passive transport was determined by performing experiments in the presence of the glucose transport inhibitor, phloretin (2 mM) in both apical and basal reservoirs. The difference between total and passive glucose transport/uptake was taken as the rate of carrier-mediated glucose transport.

### Primary syncytial cells

Cytotrophoblast cells were prepared as described previously [28], [29]. Cells were plated at a density of 0.4×10^6^ cells/cm^2^ in 6-well plates and incubated in keratinocyte growth medium (KGM; Lonza, Walkersville, MD) containing 10% FBS under a humidified air/5% CO_2_ atmosphere. Purity and syncytialization were tested as described previously [30] (data not shown). Medium was changed every 24 hr. and at 66 hr. medium was switched to serum-free DMEM containing 5 mM glucose and 0.5% BSA for 18 hr. prior to incubation in the same medium without or with 200 ng/mL IGF-I for a further 24 hr.

### Immunoblotting

To determine GLUT1 protein expression, slot-blotting was performed on membrane samples as described previously [4] using a polyclonal anti-GLUT1 antibody (1∶1,000). The membrane was washed with TBS containing 0.05% Tween-20 (TBST; 2×15 min, 2×5 min) then incubated for a further 30 min with an HRP-coupled anti-rabbit IgG secondary antibody (1∶40,000) at room temperature. Detection and visualization were performed using chemiluminescence on a Bio-Rad ChemiDoc MP imager. GLUT1 displayed a single band on Western blots as demonstrated previously [4]. Band densities were measured using Image J (NIH).

### Other assays

Protein concentration was measured by the Bradford assay [31] and glucose concentrations were measured using a hexokinase-glucose-6-phosphate dehydrogenase coupled enzyme assay [32].

### Data analysis

All data is presented as mean ± SEM. BeWo GLUT1 expression and glucose transport, syncytial and explant GLUT1 expression data were analyzed by paired t test (two-tailed) or ANOVA (Dunnett’s test). Perfusion GLUT1 expression was assessed by a one-sample t test. Glucose transport in the perfusion model was analyzed by repeated measures ANOVA with a linear trend post hoc test.

### Materials

Insulin-like growth factor I (IGF-I) was obtained from Life Technologies (Grand Island, NY) and the anti-GLUT1 antibody was obtained from Thermo Scientific (Waltham, MA). Protease inhibitor and the HRP-labeled anti-rabbit IgG antibodies were obtained from Sigma Chemical Company (St Louis, MO). [^3^H] 2-deoxy-D-glucose was obtained from American Radiochemicals (St Louis, MO). [^3^H] 3-O-methyl-D-glucose and [^14^C] L-glucose were obtained from New England Nuclear (Boston, MA). Nitrocellulose membranes (Hybond ECL) were obtained from GE Healthcare and chemiluminescence kits (Super Signal Western Pico) were obtained from Pierce Biotechnology (Rockford, IL. Permeable supports (Transwell) for growing BeWo cells were obtained from Corning (Pittsburgh, PA). All other reagents were obtained from Sigma Chemical Co. (St. Louis, MO) or Bio-Rad (Hercules, CA).

## Results

### IGF-I and GLUT1 expression in BeWo cells

**Figure 1:**
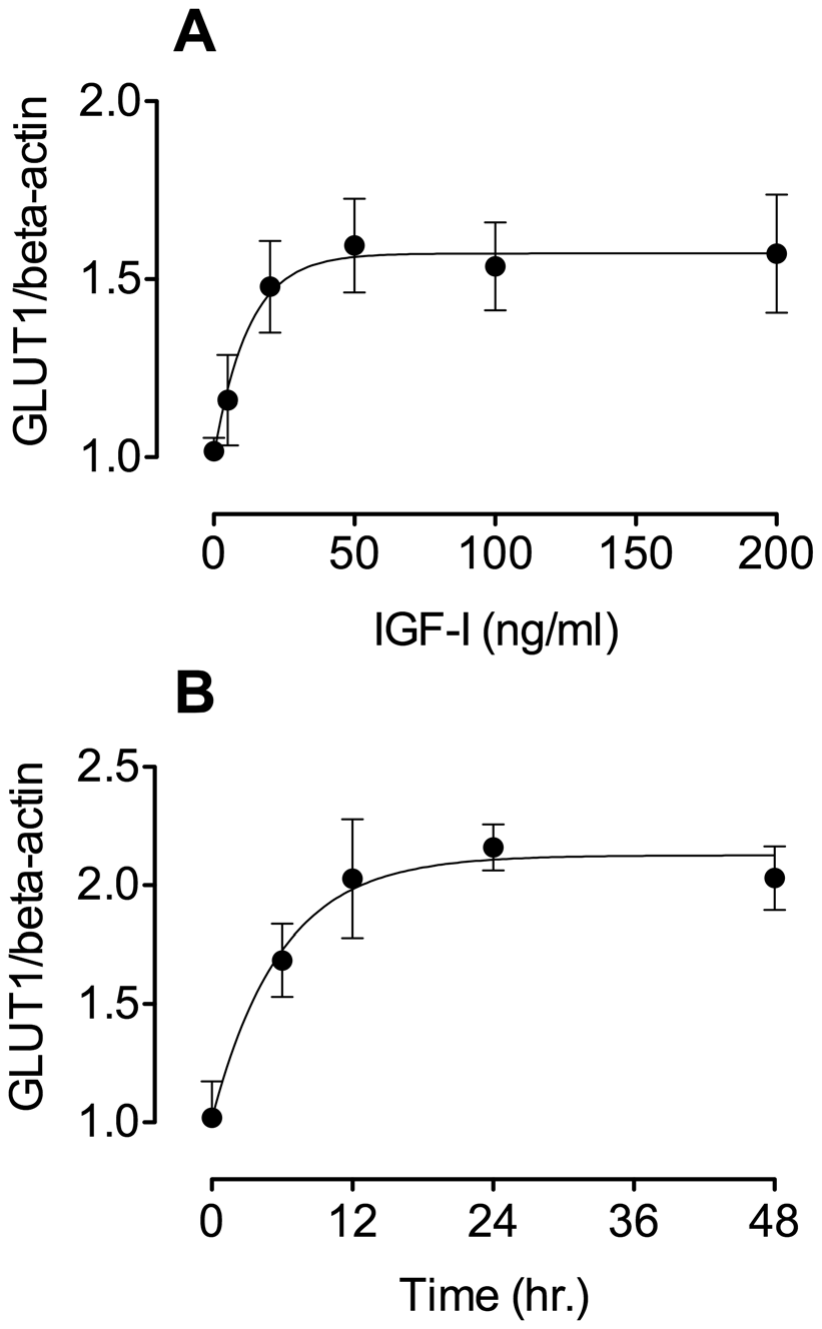
IGF-I effects on BeWo cells. (A) *Dose-response*: Serum-starved BeWo cells were treated for 24 hr. with IGF-I at doses ranging from 5 to 200 ng/mL. Extracted cell samples were used to measure GLUT1 by slot blotting. The graph shows that treated BeWo cells demonstrate a GLUT1 dose response to IGF-I terminating at a level 1.6-fold greater than control; (p<0.05, ANOVA, n = 3). (B) *Time course*: Serum-starved BeWo cells were treated with IGF-1 (200 ng/mL) for times ranging from 6 hr. to 48 hr. Extracted cell samples were used to measure GLUT1 by slot blotting. The graph shows that increased levels of GLUT1 were observed by 12 hr. with maximal levels achieved by 24 hr. (p<0.01, ANOVA, n = 3).

Initial experiments were conducted to examine the IGF-I/GLUT1 dose response and time course. For dose response experiments BeWo cells were treated with IGF-I (0–200 ng/mL) for 24 hr. following serum-free incubation. GLUT1 protein expression was measured by slot blotting after extraction of the cell protein. GLUT1 protein expression, normalized to ß-actin and adjusted to a value of unity in the absence of IGF-I, is shown in Figure 1A. GLUT1 shows an increasing and saturable response to increasing doses of IGF-I, reaching a maximum well below the dose of 200 ng/mL used in the rest of the experimental procedures. The IGF-I concentration (200 ng/mL) used in the remainder of these studies (with the exception of the perfusion studies) was chosen as a level that produces maximal stimulation and is an approximation of the normal maternal concentration of circulating IGF-I. We chose to perfuse the fetal circulation with an IGF-I concentration of 100 ng/mL, closer to that of the circulating fetal level. The actual IGF-I concentrations to which the syncytial microvillous and basal type 1 IGF receptors are exposed in vivo is difficult to assess, given the presence of multiple IGF-I binding proteins (both soluble and membrane-bound) as well as competing ligands such as IGF-II.

The time course of GLUT1 response to treatment with IGF-1 (200 ng/mL) is shown in Figure 1B. This data shows that significantly increased levels of GLUT1 were observed by 12 hr. following the initiation of treatment (p<0.01, ANOVA; n = 3). GLUT1 was maximally increased by 24 hr. with no further changes as a result of a further 24 hr. of incubation. In view of these results we chose to perform the remainder of the experiments over a 24 hr. time period to enable similar conditions for BeWo, primary syncytial and explant experiments while ensuring that effects observed were maximal.

### IGF-I effects on BeWo transport

**Figure 2:**
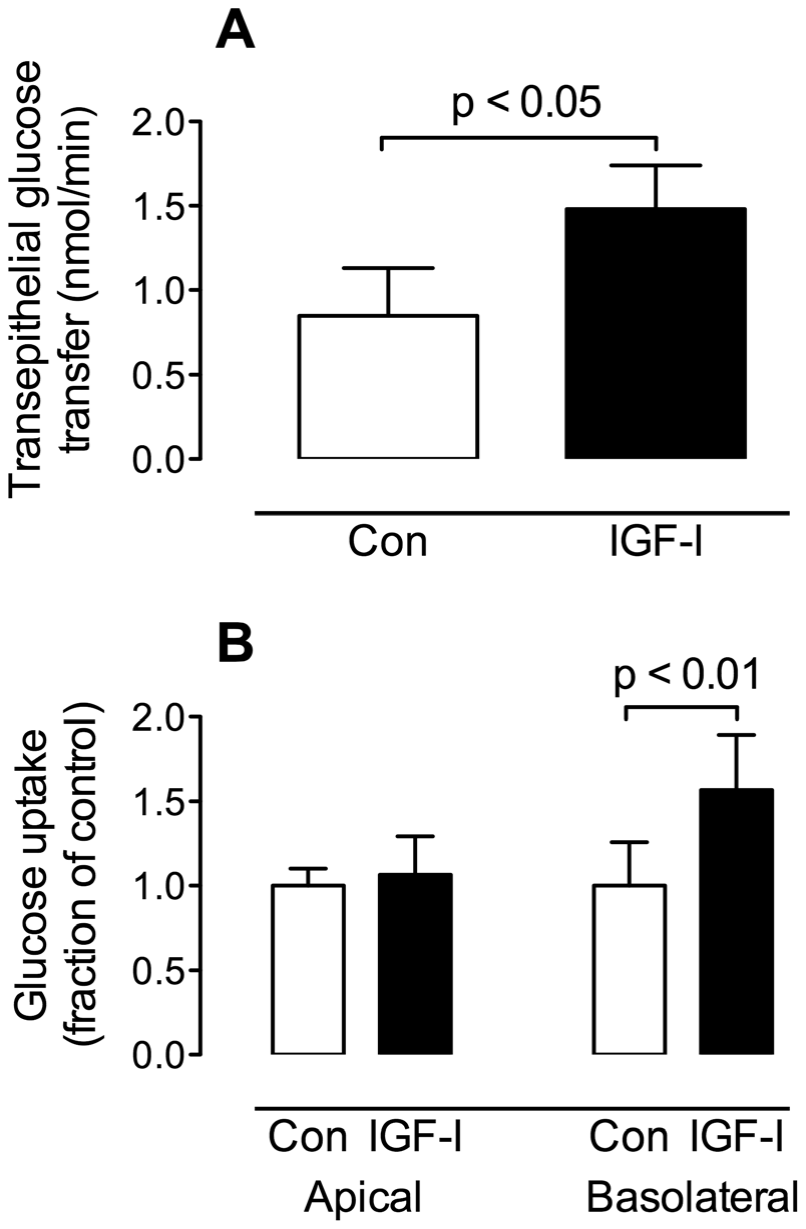
GLUT1 transport and IGF-I in BeWo cells. (A) *Transepithelial transport*: BeWo cell monolayers plated on permeable inserts were treated for 24 hr. with 200 ng/ml IGF-1 following serum-starvation. Measurements of the transepithelial transport of glucose were performed in the presence and absence of 2 mM phloretin (see Methods). Transcellular transport was calculated as the phloretin-inhibitable fraction of transepithelial transport. The results showed that treated cells had an increased rate of transcellular glucose transport (p<0.05, paired t test, n = 4). (B) *Cellular uptake*: BeWo cell monolayers plated on permeable inserts were serum starved then treated for 24 hr. with 200 ng/mL IGF-1. Following treatment, uptake of [^3^H] 2-deoxy–D-glucose into the cells across the apical or basolateral face was measured. The rate of glucose uptake across the apical face was not altered by IGF-I treatment. The rate of uptake across the basolateral face was increased by IGF-I treatment (p<0.01, paired t test, n = 4).

BeWo cells plated on permeable Transwell inserts were treated with IGF-I (200 ng/mL, 24 hr.) following serum-free incubation. Transepithelial transport of glucose across the BeWo monolayer was measured as the phloretin-inhibitable rate of appearance of glucose in the lower (”fetal side”) reservoir following transfer from the upper (“maternal side”) reservoir. Figure 2A shows that the rate transfer of glucose across the monolayer was increased 2-fold by IGF-I, from 0.85±0.28 nmol/min to 1.71±0.26 nmol/min (p<0.05, paired t test; n = 6).

Using BeWo cells plated on permeable Transwell inserts, glucose uptake into BeWo cells was measured from the upper reservoir, across the apical membrane or from the lower reservoir, across the basolateral membrane using a radiolabeled tracer ([^3^H] 2-deoxy-D-glucose). Uptake was measured in serum-starved control and IGF-I treated cells. Figure 2B shows the results of these experiments, demonstrating that while uptake across the apical membrane was unchanged by IGF-I treatment, uptake across the basolateral membrane of the BeWo cells was increased by almost 60% in cells treated with IGF-I (p<0.01, paired t test; n = 4).

### IGF-I and GLUT1 expression in primary syncytial cells

**Figure 3:**
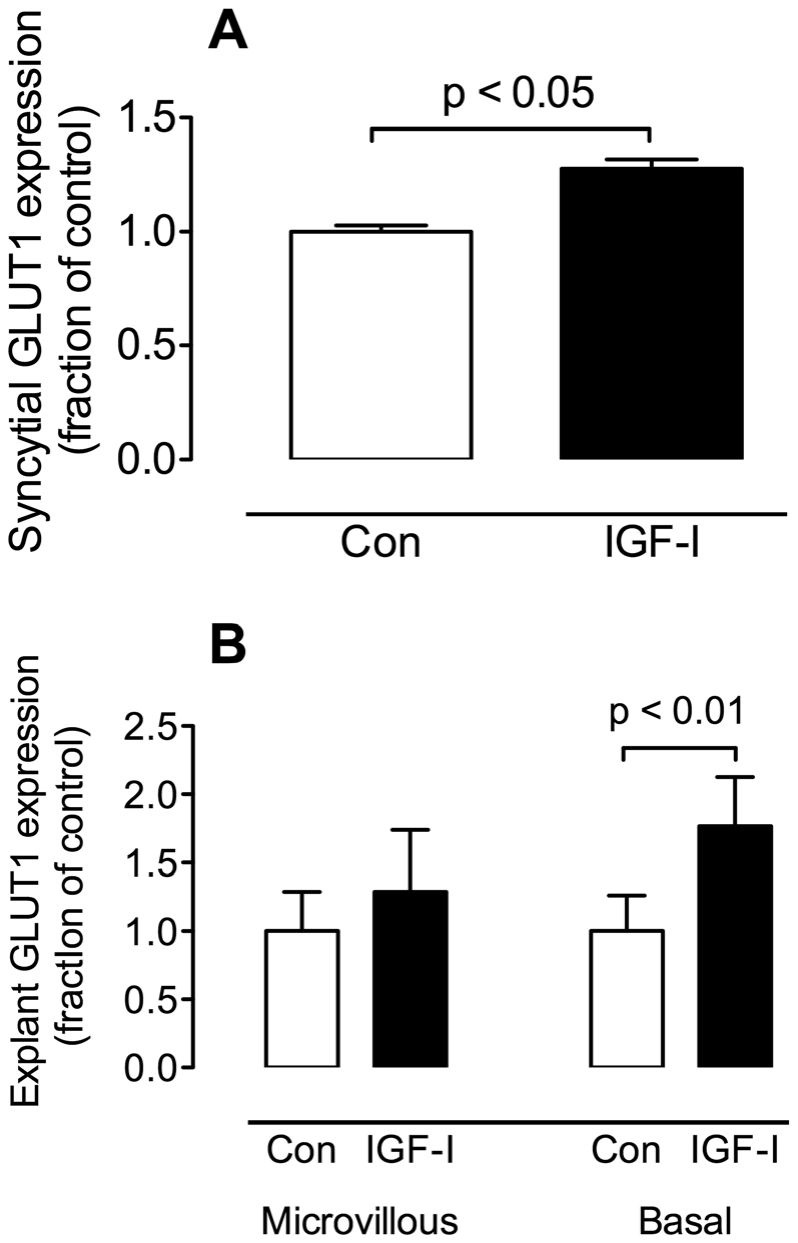
IGF-I and GLUT1 expression in primary syncytial cells and placental explants. (A) *Syncytial cells*: Serum-starved syncytial cells were treated for 24 hr. with 200 ng/mL IGF-1. GLUT1 measurement in cell extracts by slot blotting showed a significant increase as a result of IGF-I treatment (p<0.05, paired t test, n = 4). (B) *Explants*: Placental explants were serum-starved for 3 hr. then incubated with 200 ng/mL for 18 hr. Following incubation the explants were processed to produce microvillous and basal membrane fractions. GLUT1 was measured in these fractions by slot blotting. There was no change in microvillous membrane GLUT1 content as a result of IGF-I treatment however there was a substantial increase in GLUT1 in the basal membrane fraction (p<0.01, paired t test, n = 6).

We compared GLUT1 expression in primary syncytial cells following incubation in the presence and absence of IGF-I. Primary cytotrophoblast cells were incubated in KGM/FBS for 66 hr. after plating to maximize syncytialization. The cells were then serum-starved by overnight incubation in DMEM/0.5% BSA and incubated for a further 24 hr. in DMEM/0.5% BSA in the presence or absence of IGF-I (200 ng/mL). Following treatment, cells were washed×3 with cold PBS and extracted with RIPA. GLUT1 expression in syncytial samples was measured by slot blotting and normalized to ß-actin expression. The results (Figure 3A) show that IGF-I treatment increased syncytial expression of GLUT1 (p<0.05, paired t test, n = 4).

### IGF-I and GLUT1 expression in placental explants

We compared GLUT1 expression in syncytial microvillous and basal membranes prepared from explants which had been incubated in the presence or absence of IGF-I (200 ng/ml) for 18 hr. Figure 3B shows the results of Western blotting analyses for GLUT1 in explant microvillous and basal membrane fractions. GLUT1 protein expression in microvillous membranes from IGF-I-treated explant preparations was not different from that in control explants. Analysis of basal membrane fraction GLUT1 however revealed that IGF-I treated explants displayed GLUT1 levels that were significantly greater than the comparable, untreated membrane fraction (p<0.01, paired t test, n = 6).

### Placental perfusion - glucose transfer

**Figure 4:**
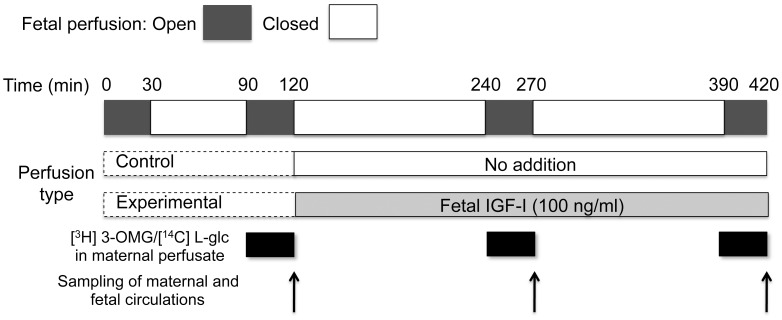
Timeline of control and experimental perfusions. A stabilization phase of 30 minutes (open fetal circulation) and 60 minutes (closed fetal circulation) was used in both control and experimental perfusions. Maternal perfusion was started 30 min after the beginning of the fetal circulation. Between 90 and 120 min the intervillous space was perfused with medium containing [^3^H] 3-O-methyl-D-glucose and [^14^C] L-glucose (0.064 and 0.032 µCi/ml, respectively) to establish rates of transfer from the maternal to fetal circulation (open fetal circulation). After the initial sampling from the maternal and fetal circulations, the fetal circulation was closed and perfusion was continued for 2 hours. During this period, the fetal perfusate in the experimental perfusions contained IGF-I (100 ng/mL) whereas the control perfusions contained no addition. After another 30-minute phase of perfusion with radiolabeled glucose on the maternal side and open circulation on the fetal side, samples were obtained from both circulations. The fetal circulation was again closed and perfusion was continued for another 2 hours containing IGF-I (experimental) or no addition (control). After a final 30 minute period of perfusion with radiolabeled glucose, perfusions were terminated following the final sampling from fetal and maternal circulations.

**Figure 5:**
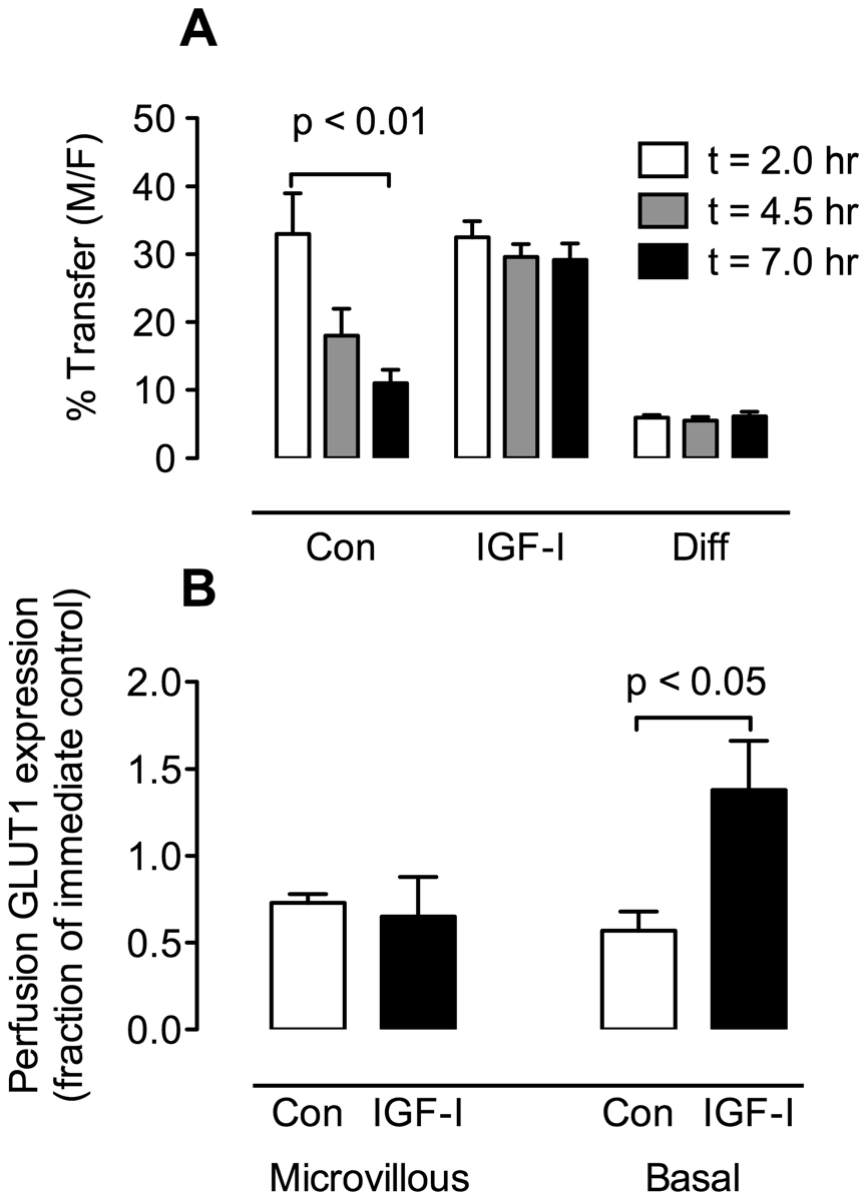
Placental perfusion. Placental perfusion was performed as described in the text, according to the protocol in Figure 4. (A) *Maternal-fetal glucose transport*. At t = 2, 4.5 and 7 hr., samples were taken from the maternal and fetal circulations and the % maternal-fetal transfer of [^3^H] 3-O-methyl-D-glucose and [^14^C] L-glucose was calculated for the control perfusions and those that were perfused with IGF-I (100 ng/mL). There was a significant linear decrease in the transfer of [^3^H] 3-O-methyl-D-glucose in the control perfusions over time (p<0.01, repeated measures ANOVA, linear trend post test; n = 4) whereas there was no change in % transfer in the IGF-I perfusions. In addition, no changes were noted in the diffusional transfer component in either the control or experimental group, shown here as the combined data. (B) *GLUT1 protein expression*. Immediately prior to perfusion, tissue samples were taken from non-perfused areas for preparation of microvillous and basal membrane fractions (immediate controls). After perfusion, the perfused lobules were dissected out and used for preparation of microvillous and basal membrane fractions. The two membrane fractions were slot-blotted to determine GLUT1 protein expression. The results show the effects of IGF-I on microvillous and basal membrane GLUT1 in the perfusions as a fraction of the GLUT1 protein expression in the respective immediate control samples. In the microvillous and basal samples from control perfusions there was a decrease in GLUT1 relative to the expression prior to perfusion. A similar result was obtained for the basal membrane from the control perfusion, however the basal membrane GLUT1 expression from the IGF-I perfusion was increased (p<0.05, one sample t test, n = 4).

We performed separate control and experimental perfusions. Both employed an open maternal perfusion circuit and the protocol shown in Figure 4 was used for the fetal perfusion circuit. The only difference between the perfusion protocols was that in the experimental group, after 120 min of initial perfusion, the fetal circuit was perfused with IGF-I (100 ng/ml) whereas the control group contained no addition. In both groups, glucose transfer from the maternal to fetal circulation was measured using a radiolabeled tracer ([^3^H] 3-O-methyl-D-glucose, 3-OMG). The percent transfer from maternal to fetal circulation was calculated from the radiolabeled tracer levels and fetal flow rate. The results in Figure 5A show that for the control perfusions the transfer of 3-OMG decreased in a linear manner over the perfusion period (p<0.01, repeated measures ANOVA, linear trend post test; n = 4). By contrast in the experimental (IGF-I-treated) group, the transfer of 3-OMG was maintained throughout the perfusion period. Diffusional transfer, measured as transfer of [^14^C] L-glucose, remained constant over the perfusion period in both groups.

### Placental perfusion - GLUT1 expression

Syncytial microvillous and basal membranes were prepared from both the control and experimental (IGF-I treated) perfused tissue. In order to control for differences in function over time following removal from the uterus, membranes were also generated from the un-perfused villous tissue of both groups, obtained at the time of the initiation of the perfusion (“immediate controls”). GLUT1 protein expression was measured by slot-blotting both the immediate control and perfused samples. The results of these measurements are given in Figure 5B, where GLUT1 protein expression in the microvillous and basal membranes of the control and experimental groups are given as a fraction of GLUT1 expression in the corresponding immediate control samples. Microvillous GLUT1 expression was reduced by 27% in the control perfusion and by 35% in the IGF-I perfusion compared to their respective immediate controls (p<0.05, one sample t test, n = 4). Basal membrane GLUT1 was reduced by 43% in the control perfusion compared to the immediate controls, but in the IGF-I perfusions basal membrane GLUT1 expression was increased by 38% compared to the immediate controls (p<0.05, one sample t test, n = 4).

## Discussion

These studies examined the role of IGF-I in the regulation of GLUT1 protein expression in trophoblast cells. We hypothesized that IGF-I would up-regulate GLUT1 expression on the basal surface, the rate limiting step in maternal to fetal glucose transport. Several different trophoblast models were used to examine the effects of IGF-I. We found that IGF-I increased GLUT1 expression in BeWo choriocarcinoma cells, associated with an increase in glucose uptake across the basolateral but not the apical surface. IGF-I treatment also increased BeWo transepithelial glucose transport. Treatment of primary syncytial cells with IGF-I increased GLUT1 expression. Administration of IGF-I to human placental explants increased syncytial basal membrane GLUT1 content compared to untreated explants, whereas microvillous membrane content was not altered. In placental perfusion studies, control perfusions demonstrated a significant decrease in the maternal-to-fetal transfer of glucose over the course of the perfusion compared to experiments in which the fetal perfusate contained IGF-I. In the control perfusions there was a decrease in basal membrane GLUT1 protein expression over the course of the experiment. In comparison, placental tissue perfused with IGF-I via the fetal circulation demonstrated an increase in basal membrane GLUT1. These results support the hypothesis that GLUT1 protein expression on the basal membrane of human trophoblast cells is regulated by IGF-I. This has significant consequences for conditions in which there are changes in circulating IGF-I concentrations such fetal growth restriction, preeclampsia or macrosomia.

Investigations of processes in the human placenta face a number of challenges as a result of the nature of tissue/cellular structures, requiring utilization of a variety of experimental models. We have used choriocarcinoma cells, primary trophoblast, placental explants and *in vitro* perfusion to examine the role of IGF-I in the regulation of GLUT1. BeWo choriocarcinoma cells model the syncytial layer *in vivo*, enabling measurement of transport properties that cannot be performed with primary syncytial cells due to the absence of a transporting monolayer. Limitations of the BeWo model are that while they are trophoblastic in nature, they are transformed cells that may respond differently compared to syncytial cells *in vivo*. Primary cytotrophoblast cells aggregate, fuse and differentiate to form syncytial cells, however current techniques do not allow for generation of a confluent monolayer, precluding the measurement of transepithelial glucose transfer or transport of glucose across the microvillous and basal membranes. Placental explants cannot be used for the measurement of transport but are comprised of the original cellular components of the tissue in their *in vivo* configuration and are therefore appropriate for examining the response of syncytial cells to external agents such as IGF-I. However previous studies have noted that syncytial degeneration begins to occur after six hours in culture [33], [34]. In these experiments we used matched control and experimental samples from the same placenta, so that any effects of syncytial degeneration on the experimental samples were also observed in the control samples. The placental perfusion model is the closest to the *in vivo* situation. It allows for measurements of both transporter expression and maternal-fetal transport of glucose, albeit under conditions which are, in part, non-physiological (e.g perfusate with a high PO_2_ but low O_2_ content). Used singly, each of the models cited above has flaws that preclude definitive conclusions. Used in combination, the commonality of results between the models gives us confidence in the results reported here.

As we noted in an earlier publication, there is very little GLUT1 present in syncytiotrophoblast which is not localized to the microvillous or basal membrane [1], and so it seems unlikely that the changes in basal GLUT1 are a result of relocalization of intracellular GLUT1. If the increase in basal membrane GLUT1 had resulted from relocalization of microvillous GLUT1 to the basal membrane, one might expect a decrease in microvillous GLUT1 concurrently with the increase in basal membrane GLUT1. The absence of a significant decrease in microvillous GLUT1 suggests that microvillous to basal redistribution is not occurring. Combined with the overall increase in GLUT1 noted in the primary trophoblast experiments, these elements point to an overall increase in GLUT1 protein expression. The increase in syncytial GLUT1 as a result of IGF-I treatment is supported by the observations of Jones et al who observed an increase in BeWo GLUT1 after transfection of cells with an adenoviral vector-human IGF-I construct [35].

Not addressed in these studies is the mode of action of IGF-I. It is quite possible that IGF-I has more than one mode of action in stimulating GLUT1 expression and activity. For example the causes of the degenerative changes in the syncytium are unknown but given the anti-apoptotic nature of IGF-I, it is possible that the differences in GLUT1 expression in the IGF-I-treated explants or in the perfusion may be related to IGF-I inhibition of (syncytial) apoptosis or other IGF-I effects promoting or supporting syncytial viability. IGF-I action to sustain tissue viability is supported by the nature of IGF-I effects on glucose transport in the perfusion experiments, where the progressive loss of glucose transfer capacity is prevented by IGF-I. While the time course of IGF-I action in the perfusion is shorter than that employed in the cell or explant experiments, we have previously shown the effects of hypoxia mimetics on GLUT1 expression over a similar time scale [36]. While not the primary purpose of these studies, it is interesting to note that addition of IGF-1 to media in experiments involving placental perfusion/explants might well improve our ability to mimic the *in vivo* maternal environment, maintain tissue viability and extend the period of experimentation.

Other verified trophoblast glucose transporters are GLUT3, GLUT8 and GLUT9 [35], [37], [38]. GLUT3 is present only on the microvillous/apical membrane [37], and given the substantial quantity of GLUT1 on this face of the syncytial cell compared to the basal membrane, it is unlikely that any effects of IGF-I on GLUT3 will alter transplacental glucose transport. GLUT8 has been described in human syncytial cells [35] but since it is localized to the late endsome/lysosomal compartments [39] it is unlikely to be involved in transepithelial transfer. While GLUT9a and 9b protein were both increased by IGF-I in BeWo cells, only GLUT9b, the syncytial microvillous form [38] relocalized to the plasma membrane [35], suggesting that IGF-I effects on this protein will not have significant effects on transplacental glucose transport.

Another aspect worthy of consideration is the location of IGF-I action. It is likely that IGF-I is able to act on type 1 IGF receptors on both the microvillous and basal/basolateral membranes in the cell models used here. It is less likely however that external, hydrophilic agents such as IGF-I are able to reach the basal (fetal-oriented) face of the syncytial cells in the cultured explants and so we must assume that the IGF-I added to explants only interacts with the microvillous (maternal-facing) surface of the syncytial cells. In the perfusion, by contrast, IGF-I was added only to the fetal circulation, thus its action was limited to receptors on the basal face of the syncytium. Despite this, our results show that IGF-I has the same effect whether it binds to receptors on the maternal- or fetal-facing membrane, increasing GLUT1 on the syncytial basal membrane. As the type 1 IGF receptor is found on both microvillous and basal membranes [13], [40], the effects of IGF-I on syncytial basal membrane GLUT1 expression can thus be mediated by changes in type 1 IGF receptor ligands in either the maternal or fetal circulation Similarly, changes in presentation of the type 1 receptor on either the microvillous or basal membrane may also influence basal membrane GLUT1 levels.

The role played by IGF-I as a growth factor is widely recognized. Equally, its clear association with fetoplacental growth has long been known. The results presented here connect these processes and provide a mechanism by which IGF-I is able to regulate fetal growth, through its up-regulation of the GLUT1 glucose transporter protein on the basal membrane of the syncytiotrophoblast. Agents such as IGF-I which alter basal GLUT1 expression will have significant effects on placental glucose transfer capacity and consequently on the levels of fetal circulating glucose. There is also strong evidence for IGF-I involvement in the regulation of placental amino acid transporters [41]–[45] and possibly fatty acid transport [46]. Experiments employing maternal nutrient restriction in the baboon showed a reduction in GLUT1 expression in syncytial microvillous GLUT1 expression, as well as reduction in amino acid transporter protein expression concomitant with inhibition of IGF-I/insulin signaling [47]. It seems plausible therefore to postulate that IGF-I plays a key role in regulating the placental transport of essential nutrients from mother to fetus. It will be crucial therefore in controlling the rate of fetal growth through regulation of the fetal nutrient supply. We have observed that reduced placental glucose transfer into the fetal circulation is associated with low fetal circulating glucose, a reduced fetal insulin level and reduced birth weight [48]. It is probable therefore that an alteration in fetal and/or maternal IGF-I, acting via changes in transporter expression, is one of the key pathways by which fetal growth is coupled to nutrient supply.
